# Reimbursement of orphan drugs in Belgium: what (else) matters?

**DOI:** 10.1186/s13023-014-0139-z

**Published:** 2014-09-12

**Authors:** Eline Picavet, David Cassiman, Steven Simoens

**Affiliations:** KU Leuven Department of Pharmaceutical and Pharmacological Sciences, Herestraat 49, PO box 521, Leuven, 3000 Belgium; Department of Hepatology, University Hospital Leuven, Herestraat 49, Leuven, 3000 Belgium

**Keywords:** Reimbursement, Orphan drugs

## Abstract

**Background:**

Most orphan drugs do not meet traditional standards of cost-effectiveness. Yet, most orphan drugs are reimbursed, which implies that other factors are taken into account at the time of reimbursement. To increase accountability of decision-makers, there is a need for more transparency in the factors that play a role in reimbursement decisions of orphan drugs. Therefore, the aim of this study is to use a combination of qualitative research methods to examine which official and non-official factors influence reimbursement decisions for orphan drugs in Belgium.

**Methods:**

Six semi-structured interviews with past or present members of the Drug Reimbursement Committee (DRC) were performed with a view to obtaining an overview of the potential factors influencing reimbursement. Additionally, these presence of these factors was assessed in the reimbursement dossiers of all orphan drugs (n = 64) for which an application for reimbursement was submitted to the National Institute for Health and Disability Insurance in Belgium between January 2002 and July 2013.

**Results:**

Different official (i.e. therapeutic value, budget impact, price and impact in clinical practice) and non-official factors (i.e. pricing and reimbursement in other countries, interference by patient organisations and experts, arguments related to quality of branded drug versus compounding, media attention, innovative character, economic importance, ethical arguments and the political climate) may have influenced past reimbursement decisions for orphan drugs in Belgium.

**Discussion:**

The identification of factors influencing orphan drug reimbursement is a crucial step in the development of a transparent and consistent framework which will guide future decision-making for reimbursement of orphan drugs.

**Electronic supplementary material:**

The online version of this article (doi:10.1186/s13023-014-0139-z) contains supplementary material, which is available to authorized users.

## Background

In Europe, rare diseases are defined as life-threatening or chronically debilitating diseases with a prevalence of 50 out of 100000 individuals or less. Legislation is in place to stimulate the development of so-called ‘orphan drugs’ for the treatment of rare diseases. Incentives, such as a 10-year period of market exclusivity, are intended to increase the commercial value of orphan drugs by reducing R&D expenses and shortening time-to-market [1]. Currently only 72 orphan drugs are marketed in the European Union [2]. Pricing and reimbursement decisions are a responsibility of the Member States of the European Union which govern different policies. As a result, availability and access to orphan drugs still differs throughout the European Union [3–7].

Because of their high prices, most orphan drugs do not meet traditional standards of cost-effectiveness. Yet, most orphan drugs are reimbursed, which implies that other factors are taken into account at the time of reimbursement [8]. Rosenberg-Yunger *et al.* conducted qualitative case studies to determine how drug advisory committees made reimbursement decisions for two orphan drugs in Canada, Australia and Israel. Reimbursement recommendations were made based on technical factors (such as cost-effectiveness) as well as other factors (such as ethical considerations). The authors concluded that insight in these other factors is necessary to improve the reimbursement of future orphan drugs [9]. Cerri *et al.* examined how different factors influence reimbursement decisions made by the National Institute for Health Care and Clinical Excellence (NICE) in the United Kingdom. The multivariate regression analysis showed that only 26 percent of the variability in decisions can be explained by four outcome variables (i.e. superiority of the primary endpoint, the incremental cost-effectiveness ratio (ICER), the number of pharmaceuticals appraised within the same appraisal and the year of the appraisal). The results suggested that decisions are influenced by the decision-making process, the clinical evidence and by the socio-economic and political context [10]. In England, drugs for very rare diseases are assessed separately taking into account broader criteria such as health gains, societal values and impact on clinical practice [11]. The Belgian application procedure for reimbursement of orphan drugs has been described in detail in a report by the Belgian Health Care Knowledge Centre [12]. Briefly, a pharmaceutical company simultaneously submits a reimbursement dossier to the Drug Reimbursement Committee (DRC) of the National Institute for Health and Disability Insurance (NIHDI) and a price demand to the Federal Public Service (FPS) Economy. As orphan drugs are considered an exception in class 1 (i.e. drug with an added value), a budgetary impact study is required in the reimbursement dossier but a cost-effectiveness analysis is not. The DRC is composed of one (non-voting) chairman and 30 members; 22 voting members (i.e. seven academics, eight representatives of the sickness funds, four representatives of the physicians’ association, three representatives of the pharmacists association) and eight non-voting members (i.e. four representatives of the Ministry of Public Health, of Social Affairs, of Budget and of Economics Affairs, one representative of the NIHDI, and two members of Pharma.be (representing the pharmaceutical industry in Belgium), and one member of Febelgen (representing the generic pharmaceutical industry)). The DRC evaluates drug reimbursement requests based on the five criteria: therapeutic value, price, proposed reimbursement tariff, the importance of the drug in clinical practice, and the budgetary impact of the drug [13]. The DRC may decide to compose a group of experts to evaluate the application. A proposal for reimbursement is prepared by the DRC within 150 days after the application. The final decision is made by the Minister of Social Affairs within 180 days, after approval of the Minister of Budget has been obtained. After a negative advice, companies may enter into negotiations for managed entry agreements (in which for example, the pharmaceutical company refunds a predefined percentage of the price for every unit sold) as defined by the Royal Decree of December 21^th^, 2001 [14]. Dupont and Van Wilder performed a qualitative analysis of Belgian reimbursement dossiers of all orphan drugs with a specific focus on the evidence related to the five reimbursement criteria (therapeutic value, price, proposed reimbursement tariff, the importance of the drug in clinical practice, and the budgetary impact of the drug) [15]. They concluded that factors other than the official criteria seem to play a role in the decision-making process, thereby creating uncertainty [13].

With a view to increasing accountability of decision-makers, there is a need for more transparency in the factors that are taken into account in reimbursement decisions of orphan drugs [11]. Frameworks for the structured analysis of reimbursement decisions have been proposed [16,17]. These frameworks allow for comparison of the decision-making process across health care systems and technologies [18]. Analysis of reimbursement decisions may reveal preferences of third party payers which deviate from pre-defined processes [19]. However, further research is needed to assess the validity and transferability of these frameworks [18]. Also, it remains a challenge to integrate concepts such as transparency and stakeholder involvement in the framework [19]. While past studies applied quantitative research methods, this study aims to obtaining an alternative view of the same issue. Therefore, the aim of this study is to use a combination of qualitative research methods to examine which official and non-official factors influence reimbursement decisions for orphan drugs in Belgium. Furthermore, we aim to document these findings with examples from Belgian orphan drug reimbursement dossiers.

## Methods

### Semi-structured interviews

Semi-structured interviews were used as they enable the interviewer to elaborate on specific aspects or insights of the interviewee or when certain aspects are unclear for the researcher. A total of seven former or present members of the DRC (both voting and non-voting members) were identified through selective expert sampling (i.e. participants with an expert opinion on the subject matter) and snowball sampling (i.e. each participant was asked to provide name(s) of possible other participants) and were contacted by e-mail to participate. Further arrangements, concerning time and place of the interview, were made by e-mail or telephone. Semi-structured interviews with six participants were conducted between January 17^th^ and February 5^th^ 2014. A semi-structured interview guide (Additional file 1: Appendix 1) was drafted in the form of a table presenting a non-exhaustive list of potential factors influencing reimbursement derived from a literature review [10,13,15,20]. Respondents were asked to comment on that list, provide real-life examples and suggest changes or additions. Respondents participated voluntarily and were not remunerated. Because of the nature of the interviews, it was not required to seek approval from a research ethics committee. The anonymity of the participants and confidentiality of the answers were guaranteed. The interviews were audio-recorded and verbatim transcribed. The interviews were analyzed using the QSR NVivo 10 software according to the five stages of the framework analysis: (1) familiarization (reading of the transcripts and notes, listening to the digital recordings); (2) identifying a framework; (3) indexing (i.e. applying the framework to the data); (4) charting; and (5) interpreting [21,22]. Appropriate quotes were selected from the transcripts and translated from Dutch or French to English as truthfully as possible. To facilitate reading, deleted parts of the quotes are indicated by a “(…)” and additional information is provided between square brackets. The coding [***I1***, ***I2***, …] identifies the interviewee.

### Analysis of reimbursement dossiers

In the context of documenting the findings from the semi-structured interviews with examples from Belgian orphan drug reimbursement dossiers, the reimbursement dossiers for all orphan drugs (n = 64) for which an application for reimbursement was submitted to the NIHDI in Belgium between January 2002 and July 2013 were analysed. In Belgium, drug reimbursement dossiers are retained by the NIHDI. The final decision made by the Minister of Social Affairs and the evaluation report of the DRC are publicly available since respectively January 2002 and April 2007. Complete dossiers consist of the application for reimbursement written by the pharmaceutical company, the first scientific report by the DRC (Day 30 report), the evaluation report by the DRC (Day 60 report), the decision on the maximum price, the final proposal of the DRC (Day 150 report), remarks and answers to the DRC’s questions by the pharmaceutical company, the advice of the Finance inspector and the approval of the Minister of Budget and the final decision made by the Minister of Social Affairs (Day 180). Examples provided in the Results section originate from these documents. The complete dossiers were consulted on-site between February 4^th^ and February 24^th^ 2014 after approval for research purposes was granted. The following information was extracted using a reporting template: orphan drug; indication; applicant; dossier; start and stop date of the reimbursement dossier; advice of the DRC; advice of the financial inspector; approval of the Minister of Budget; final decision; therapeutic value; clinical evidence; applicability and user-friendliness; medical and social need; price; budget impact; availability in other countries; special patient characteristics; patient organisation; employment, research and infrastructure in Belgium; support for research and innovation; other peculiarities.

## Results

The following provides an overview of the different factors which may have an influence on reimbursement of orphan drugs in Belgium. The DRC evaluates orphan drug reimbursement requests based on official factors such as therapeutic value, budget impact, price and importance of the drug in the clinical practice. It is however conceivable that other, non-official factors have an influence on the decision-making by the DRC and the Minister of Social affair. Each factor is documented with quotes from members of the DRC and/or examples from Belgian orphan drug reimbursement dossiers reflecting the views of the DRC on the one hand and the applicant on the other.

### Therapeutic value

Demonstrating therapeutic value for orphan drugs is often complicated, for instance due to the use of surrogate endpoints, the small study population and the heterogeneous presentation of rare diseases [23]. Furthermore, it is often unclear how improvement in a hard or surrogate endpoint translates into clinical benefit for patients thereby complicating reimbursement decisions.

*“There are also a few [orphan drugs] for which the evidence is far less convincing, I’m thinking of some enzyme replacement therapies”. [****I1****]*

*“The evidence for effectiveness is often not there”. [****I2****]*

*“Clinical progression, that’s an important factor! We want to know, does the drug work? Statistical significance is often a problem, because a lot of studies lack power due to the number of patients. But also, is it clinically relevant?” [****I3****]*

### Cost-effectiveness

No cost-effectiveness analysis is required for orphan drugs, as such, the DRC does not have access to pharmaco-economic data.

*“We don’t have a pharmacoeconomic evaluation for orphan drugs, we have no QALY, nothing!” [****I4****]*

### Budget impact

A budget impact analysis is required and is thoroughly inspected by the DRC.

*“The situation is like this, experts at the NIHDI check the budget impact [provided by the pharmaceutical company] and the parameters used to calculate that budget impact, for example, if the firm says that there are only 400 patients and our experts say that there are in fact 2000, that will have a drastic effect on the budget impact, so we need to find a balance”. [****I5****]*

For example, the original budget impact for Xyrem® amounted to €614,016, based on 52 treated patients. The DRC however expected the number of patients to be at least fourfold (250), resulting in a budget impact of €2,460,000. In other cases, the DRC was of the opinion that additional expenses also need to be taken into account in the budget impact analysis. For example, each patient treated with Soliris® needs a meningococcal vaccination (costing approximately €27 per vaccine), which was not taken into consideration in the initial calculations.

*“The estimation of budget impact is extremely important, on the one hand to make the decision right now, but on the other hand, in view of the future, we can accept some deviations here and there, but hopefully, there will be deviations in both directions, both up and down”. [****I2****]*

For instance, in 2008 the budget impact for Yondelis® was estimated to amount to €443,044 for the treatment of soft tissue sarcoma. In 2012, the budget impact turned out to be much higher, (€3,177,766). Afterwards, the ex-manufacturer price of the drug was reduced by five percent, which should reduce the budget impact to €2,960,089.

*“We have to be careful, we have a certain budget, and we try not to surpass those budgets, because that is at the expense of other things”. [****I5****]*

Some pharmaceutical companies attempt to highlight the low(er) budget impact. To that end, the applicant of Ilaris® added a comparison between the relatively low budget impact of Ilaris® and other expensive orphan drugs (Aldurazyme®, Elaprase®, Myozyme®,…). A similar overview was provided for Cystadane®. Additionally, it was stated that the one-year budget impact for Cystadane® would only represent 0.0011% of the total drug expenditure by the NIHDI.

### Importance in clinical practice

The importance of a drug in clinical practice takes into account factors such as therapeutic need (dependent on patients’ age, mortality, disease burden,… ), place of the drug in the clinical practice (availability of alternative treatments) and adequacy of the package size. The age and disease burden of patients is taken into account in reimbursement decisions. Some interviewees acknowledge difficulties in refusing reimbursement for young patients with life-threatening diseases.

*“I guess, if you have two options, approve or not approve, then it is hard, knowing that there are only 5 children affected, not to approve the drug, because that’s no longer a whole group of patients, but a very specific situation”. [****I1****]*

*“Something like that [disease burden] always gets taken into account implicitly, never explicitly, in the decision”. [****I3****]*

*“The age of patients, hmm, I guess. I think there is a tendency to treat children rather than adults, someone at the age of for example 60 years… There is a tendency to treat younger people, to give them life expectancy, rather than adults who, in that stadium, often have a less debilitating disease”. [****I3****]*

In some cases, an age limit is imposed because the registered indication is restricted to a selected age group.

*“There was a drug, a long time ago, for the treatment of multiple sclerosis, and was registered for use in patients up to the age of 51. Therefore, it was only reimbursed up to that age, … but we gave that up, because an upward age limit is very delicate. But of course there is no clinical evidence above that age”. [****I2****]*

The DRC suggested limiting the reimbursement of Adcetris® to patients below the age of 65. This was deemed unethical by the pharmaceutical company, because Adcetris® is the only option for these patients (as they are less likely to qualify for stem cell transplantation). Similarly, the DRC suggested excluding patients below the age of five from reimbursement of Aldurazyme®. The applicant was of the opinion that young patients with the highest treatment needs were excluded in that way.

The drugs’ place in clinical practice is also considered.

*“If there is no other therapy, and this new one potentially has some benefit, then it isn’t difficult, but if there is an alternative, then the question is, has the alternative been registered officially?” [****I2****]*

Comparisons to alternative treatments are often made in applications for reimbursement. For example, the application for Tracleer® specifically mentions that Tracleer® is the only oral treatment, suggesting an increased user-friendliness. The application for Sprycel® mentions that the use of the alternative treatment (Glivec®) can lead to the emergence of resistance. Increasing dosages of Glivec® are associated with more secondary effects and limited effectiveness. Therefore, Sprycel® would constitute a viable alternative. The application for Vpriv® specifically mentions that there were shortages of Cerezyme® (the alternative drug) in 2009 and 2010, emphasizing the need for an alternative treatment for Gaucher’s disease. Finally, there are some cases in which the use of the new drug has been established in other countries. For example, the application dossier for Revlimid® mentions that the drug has already been included in treatment guidelines from the National Comprehensive Cancer Network, the British Committee for Standards in Haematology and the UK Myeloma Forum.

Lastly, the DRC also takes into consideration the relevance of the package size as a function of the intended therapy. Inadequate package size can lead to wasting, as voiced by one of the interviewees.

*“For some orphan drugs there are important rest fractions. If for example dosing is based on mg per kg [body weight], and you don’t need the second half of the flacon, you will have to throw it away”. [****I3****]*

For example, the DRC judged that the adequacy of 10 mg vials of Trisenox® is not suited to administer a daily dose of 0.15 mg/kg because more than three quarters of patients weigh more than 66 kg and would therefore need two vials per day. Similarly, the DRC feared that the package of Mepact® (4 mg in 50 ml) would inevitably lead to important wasting. The applicant mentioned that the current dosage of Replagal® is better suited for dosage titrations in patients with low body weight as opposed to its alternative Fabrazyme®. In other cases the dosage form was problematic.

*“[about Siklos*®*] And they were big tablets, for kids of two or three years of age, totally unsuitable … and not even in a malleable form, or as a syrup”. [****I2****]*

### Price

The price of an orphan drug is often an issue of debate, for instance if the price of the orphan drug is high compared to the price of the pharmaceutical compounding (e.g. Lysodren® versus mitotane, Firdapse® versus 3,4 DAP) or the price of the same drug for a more common indication (e.g. Afinitor® versus Certican®, Revatio® versus Viagra®). Unofficial prices are not communicated, as it could have a negative impact on parallel trade. For example, a request from the DRC to lower the price of Vyndaqel by 30%, was turned down by the pharmaceutical company because lowering the Belgian price would lead to parallel export and would thereby endanger the availability of the drug in Belgium.

*“I think it’s a good thing we are carefully moving away from the axiom “we don’t change the price of orphan drugs”. But we can’t do that, as long as there is no transparency in price setting. (…) I think it’s OK to discuss the price of orphan drugs; this [current situation] is no longer reasonable and sustainable. (…) I can understand that, with parallel import and export, it makes companies are not eager to communicating unofficial prices, but it also makes price setting very vague”. ****[I2]***

After a negative reimbursement advice, the pharmaceutical company may enter into negotiations for managed entry agreements (MEAs). MEAs are intended to balance the need for access to potentially beneficial orphan drugs with the uncertainty related to cost, safety, effectiveness, [24]…

*“Companies are open to that [managed entry agreements], because it’s important for them to come on the market at a certain price”. [****I3****]*

MEAs are not part of the public domain. Only few proposals for MEAs were found in the reimbursement dossiers, for example in which the pharmaceutical company suggests to provide a number of units for free (e.g. Prialt®), to refund a predefined percentage of the price for each unit sold if the budget exceeds a certain threshold (e.g. Votubia®), or to refund a percentage of the total turnover (e.g. Firazyr®). More details are not communicated.

### Price and reimbursement in other countries

Pricing and reimbursement conditions in other countries are considered during the reimbursement decision. Furthermore, these results suggest that other countries are doing the same.

“*Countries are finding each other in terms of reimbursement, and we are not the difficult ones I guess, not at all”. [****I2****]*

*“We always make a list of that, the price and reimbursement conditions in other countries”. [****I3****]*

*“In certain cases, we take that into account, yes yes yes, more and more, certainly related to price, we look at what price is reimbursed elsewhere. (…) We take a look at what’s happening in Europe (…) The official price éh, we only see the official price”. [****I4****]*

For example, in the application dossier for Vpriv® it is mentioned that a higher price than the one requested in Belgium is fully reimbursed in the UK, Germany, Switzerland, The Netherlands, Slovenia, Denmark and Norway. Similarly, the application dossier for Evoltra® states that the drug is already available, without restrictive prescribing rules and at the same price in inter alia UK, Ireland, Spain, France, the Netherlands. The DRC issued a negative advice for the reimbursement of Wilzin®, and pointed out that a similar negative assessment was also given by the Dutch Health Care Insurance Board (CVZ).

### Patient organizations

Officially, patient organizations are not consulted during the decision-making process, but they can play an important role in creating awareness about disease-specific problems and in understanding the impact of the drug in daily life.

*“They [patient organizations] are not consulted, but they alert us of the different problems they experience, so we can understand the problem. So they have a role when it comes to creating awareness about the problem”. [****I5****]*

*“In those cases [when clinical impact is unclear], patient organizations or doctors can play an important role, because we, as members of the DRC not always value the importance of clinical impact. For example, it they say that patients are able to walk ten meters instead of five, then yeah, is that worth €200,000 a year? But if someone tells you that because of that five extra meters, he can now be independent, go to the toilet, do small domestic work, … well that changes at a lot”. [****I3****]*

*“Pharmaceutical companies sometimes also add a letter from the patient organization in the dossier. We have dossiers with letters from patients stating that the drug is important to them”. [****I4****]*

For example, a letter from a patient organization, highlighting the user-friendliness of the drug, was added to the application dossier for Tobi Podhaler®. Similarly, a handwritten letter by a patient describes her experiences with other treatments and Exjade® and emphasizes the medical and social need for this adapted form of iron chelation.

One interviewee had specific remarks.

*“It’s not always more objective with patients there. (…) To explain the practical course of something it can help, but it’s definitely not always necessary. (…) Everyone [in the DRC] signed a conflict of interest form, but patients and patient organizations should do the same, because there is a need for more transparency, how do most patient organizations get funded?” [****I2****]*

### Expert opinion

In the same way, letters from experts are sometimes added to the application dossier with a view to confirming the medical need and showing their support. For example a list of arguments from Belgian experts, highlighting the added value (i.e. the ease of dose adaptation) of Siklos®, was added to the application dossier. Similarly, eight neonatologists signed a letter requesting reimbursement of Pedea® because it would have a substantial added value in the treatment of patent ductus arteriosus.

### Quality of the branded drug versus the pharmaceutical compounding

Pharmaceutical companies may argue that the quality of the branded drug is higher than that of the pharmaceutical compounding. The application dossier for Pedea® specifically mentions that all components of the drug are in agreement with the requirements from the European Pharmacopoeia. They argue that in pharmaceutical compounding, there is no quality control. Human errors can be fatal, as illustrated by some newspaper articles added to the application dossier. In another case, the applicant argued that the pharmaceutical compounding of 3,4-DAP is not a suitable alternative for Firdapse®. An article from a Dutch journal was added to the dossier for Wilzin® which stated that more than a quarter of pharmaceutical compounding in hospitals did not meet the minimum quality requirements. Additionally, there was mention of a Dutch lawsuit in which a Wilson’s disease patient took legal action against his doctor and pharmacist due to chronic under-dosing. Even so, the DRC appears not susceptible to these arguments, often because the price of the pharmaceutical compounding is significantly lower. For example, there is a five-fold difference between the daily price of zincacetate and Wilzin®.

### Media attention

Rare diseases and orphan drugs often appear in the media. Unfortunately, the communication about the development of upcoming orphan drugs is oversimplified and overly positive which could influence the members of the DRC in the decision making process.

*“The communication about drugs in development is very bad, without any nuance. If a trial has just started, it’s like it has already succeeded, and people only hear that part of the story and cherish illusions, think they are being fooled, or don’t get what they deserve to have, but in fact, it’s not even there yet”. [****I2****]*

*“And there is another group, the academics, which are often forgotten! There are those who are forced by the firms, but there are others who, without pressure, out of pride for their own work, and also eagerness, start to communicate about it [new treatment] way to soon. You often see announcements that they found something in Leuven or Ghent [location of Belgian universities] for the treatment of this or that, but most of the time, it’s yeaaars before registration is even possible. And the media of course jump on it, but those academics have a responsibility too, because they give hope to patients that not always should be hoping”. [****I4****]*

However, interviewees believe that media attention has no direct influence on the decision-making process.

*“Well indirectly, it [media attention] creates awareness about the problem, which is good and important, but I think there is a big difference between being aware and being influenced in the decision making”. [****I1****]*

*“The timing [of media attention] is often not in line with the DRC, in general, if something happens, … because the media, it’s created, either too soon, long before a dossier is opened, and then a process is forced because “we have to do something”, and then the DRC leaves the decision to the Minister. Or when something has gone wrong, or is about to go wrong, well yeah “wrong”, … a negative decision”. [****I6****]*

In 2013, the reimbursement of Soliris® for the treatment of atypical hemolytic uremic syndrome was refused. Controversy arose when the case of an aHUS patient was shown in the media. Afterwards, it became clear that the pharmaceutical company deliberately brought attention to this case in the media with a view to influencing the reimbursement decision. Soliris® was later approved for reimbursement, by the Minister, after an unknown price reduction, thereby overruling the negative advice from the DRC. This may serve as an example in which media attention might have influenced the decision-making.

### Innovative character

Pharmaceutical companies may argue that the innovative character of the drug may warrant reimbursement. The application dossier for Firazyr® mentions that the drug recently has been pronounced “the year’s best idea in health” by a leading medical journal. Yet, according to interviewees, the level of innovativeness of a new drug is not considered in reimbursement decisions.

*“What is meant by innovation? Everyone can say, it’s innovative, but in what area? Is it something new, a drug for something without [therapeutic] options? Or is it something for which we have an alternative, but with less side effects, or more user-friendly? Ok, those are also innovations, but we also have to look, does the innovation justify the price? It’s a balance between all factors”. [****I5****]*

*“If you have a new method of action, then it is innovative, but it’s not because it’s innovative that it actually works, I mean clinically works. Innovation is perceived by the general public as something that has a positive effect on life expectancy or quality of life”. [****I3****]*

### Economic importance

Investments made by the government or the pharmaceutical company in the development of the drug may well be considered in reimbursement decisions.

*“In my experience, if research is supported by the government, to support the development of orphan drugs, that’s good, but it also leads to the expectation that reimbursement is the icing on the cake”. [****I1****]*

*“I remember that, in a few dossiers, employment in Belgium was specifically mentioned by the company”. [****I1****]*

*“I think that companies often mention that their company has research and development in Belgium, which creates employment, and saying there are many employers in Belgium. (…) What they are basically saying is, “do you want to make it more difficult for us than other countries?” [****I2****]*

For example, it is stated in the reimbursement dossier for Myozyme® that the marketing of a 200 mg vial (which is better suited for dosage titration) will depend on the successful production of the drug in Geel, Belgium.

*“The support is not only for innovation or logistics or infrastructure, but also for whole departments, and specialized nurses. There are also investments in fundamental research … and in training of doctors”. [****I4 and I6****]*

For example, the drug Gliolan® may only be used by neurosurgeons that have completed a training course. The application dossier mentions that ten Belgian doctors already completed the course prior to the application for reimbursement.

### Ethical arguments

Ethical arguments, such as the rule of rescue, right to treatment and equity in access to treatment often surface during complex reimbursement decisions [25–27]. Interviewees expressed difficulties they encounter when balancing these ethical arguments.

*“It’s difficult, certainly if it involves children with life-threatening diseases, to keep the discussion purely scientific (…) As a doctor, it is not always straightforward (…) on the one hand you want to leave “no stone unturned” for each individual patient, but on the other hand, we have to look at the bigger picture and balance some things. (…) I think maybe that is the core of the problem, because it’s about individual patients, children or adults, if it’s about larger groups than it remains anonymous and easier to judge objectively. We are not well trained to make those kinds of decisions”. [****I1****]*

*“We had a discussion in the DRC once, in which we said, this [decision about the reimbursement of an enzyme replacement therapy for children] is in fact an ethical discussion, and we are not an ethical committee”. [****I2****]*

### Political climate

In the final reimbursement decision, the Minister of Social Affairs can deviate from the advice of the DRC out of social and/or budgetary reasons.

*“The Minister can deviate [from the advice of the DRC] out of social or economic reasons. (…) But that is a wide concept, it’s not defined, and we don’t know it either”. [****I3****]*

Interviewees are unclear as to the influence of the political climate on the decision-making.

*“I think that it [upcoming elections] plays a role, but … we have so many elections, almost every year is an important election year”. [****I2****]*

*“You can’t expect an answer from us here, but honestly, I would be surprised”. [****I6****]*

## Discussion

This study provides an analysis of which official (i.e. therapeutic value, budget impact, price and impact in clinical practice) and non-official factors (i.e. pricing and reimbursement in other countries, interference by patient organisations and experts, arguments related to quality, media attention, innovative character, economic importance, ethical arguments and the political climate) may have influenced reimbursement decisions for orphan drugs in Belgium. To that end, we provided an overview of relevant quotes made by (current or past) members of the DRC and documented how these different factors were put forward and interpreted by both applicant and the DRC in the reimbursement dossiers.

Our qualitative results confirm previous quantitative findings from the international literature. Dakin *et al.* found that the number of randomized controlled trials and the presence of a submission from a patient organization are predictors of a positive evaluation by NICE [20,28]. The study from Denis *et al.* concluded that evidence of therapeutic value tended to be very limited and often derived from few clinical studies [13]. Dupont and Van Wilder found that the same quality of clinical evidence as required for medicines for common indications was not required for orphan drugs. Furthermore, with respect to dosing, they uncovered that especially for metabolic diseases, dose-finding studies were lacking [15]. The reimbursement of Cerezyme® was initially refused in Israel due to high cost. Currently, researchers are trying to establish whether lowering the dose to less than a quarter of manufacturer’s dose is feasible (i.e. without a change in effectiveness with a view to significant cost savings [9]. Finally, Cerri *et al.* found no significant association between whether or not the decision by NICE was made in an election year [10]. From this, it appears as if similar non-official factors play a role in the reimbursement of non-orphan drugs, although no comparison has been made between orphan and non-orphan drugs.

Multi-criteria decision analysis (MCDA) has been proposed as a framework to evaluate new (orphan) drugs for reimbursement based on a combination of several criteria. In an MCDA, the relevant criteria and their importance are defined a priori. Criteria such as availability of existing treatments, treatment safety, manufacturing complexity have been proposed [11,29]. These proposed criteria are however not exhaustive. Some factors identified in this study might be added to an MCDA as a measurable criterion, for example, if the drug creates employment and if the drug is reimbursed in other countries. Other factors such as media attention or political climate are less or even non quantifiable. Weights should be attributed to each of the criteria based on the values attached to them by society [29]. In any case, transparency is needed to enhance consistency in the decision-making process [11].

This study provides insight into the different factors that might be taken into consideration during reimbursement decisions for orphan drugs in Belgium. Because of the qualitative nature of the study, we are unable to quantify the effect of each factor on reimbursement decisions. Furthermore, it is most likely that there is interplay between the different factors.

## Conclusion

This study has shown that reimbursement of orphan drugs in Belgium is possibly influenced by a combination of official and non-official factors. The identification of these factors is a crucial step in the development of a transparent and consistent framework which will guide future decision-making for reimbursement of orphan drugs.

## Additional file

Additional file 1:
**Appendix 1.** Semi-structured interview guide.

## Abbreviations

DRC: Drug reimbursement committee
ICER: Incremental cost-effectiveness ration
PFS: Federal public service
NICE: National institute for health care and clinical excellence
NIHDI: National institute for health and disability insurance
MCDA: Multi-criteria decision analysis
