# Supplementary material for: Reimbursement of orphan drugs in Belgium: what (else) matters?
Source: Orphanet J Rare Dis. 2014 Sep 12;9:139. doi: 10.1186/s13023-014-0139-z (PMC4172832; doi:10.1186/s13023-014-0139-z)
Supplement: Additional file 1: — Appendix 1. Semi-structured interview guide. [file 13023_2014_139_MOESM1_ESM.docx]

Appendix 1: semi-structured interview guide

**Introduction:**

Dupont et al.: “lower quality of clinical evidence, a higher level on uncertainty on the clinical effectiveness, safety and incremental cost-effectiveness, and a higher budgetary impact are accepted for orphan drugs”

Denis et al.: “Reimbursement was awarded to the majority of orphan drugs. In addition to the official criteria, other negotiable factors, such as price adjustments, employment incentives, patient population restrictions and funding of diagnostic tests by the company, seemed to play a role in the reimbursement decision. Despite the low number of patients, randomized controlled trials were conducted for many orphan drugs. Budget-impact analyses were simplistic and did not consider the impact across multiple indications. Some differences were also observed between the clinical evidence submitted to the EMA and that submitted to the Belgian DRC. In addition to the official criteria, **other negotiable factors, such as price adjustments and employment incentives**, may play a role in Belgian reimbursement decisions of orphan drugs.”

**Disscusion - factors playing a role in reimbursement of orphan drugs in Belgium:**

|  | **Factors** | **How does this become evident? Possible examples?** |
| --- | --- | --- |
| **Official factors** | Therapeutic value |  |
|  | Price |  |
|  | Proposed reimbursement tariff |  |
|  | Importance in clinical practice |  |
|  | Budgetary impact |  |
| **Other factors** | Innovative character |  |
|  | Media attention |  |
|  | Influence of patient organization(s) | for example, presence of a patient organization in Belgium |
|  | Political climate |  |
|  | Economic importance (employment in Belgium) | for example, presence of a company branch in Belgium |
|  | Ethical arguments |  |
|  | Age of patients |  |
|  | Price and reimbursement in neighboring countries |  |
|  | Cost of compounding |  |
|  | **Others?** |  |
